# Supplementary material for: Impact of Adipose-Derived Mesenchymal Stem Cells (ASCs) of Rheumatic Disease Patients on T Helper Cell Differentiation
Source: Int J Mol Sci. 2022 May 10;23(10):5317. doi: 10.3390/ijms23105317 (PMC9140468; doi:10.3390/ijms23105317)
Supplement: Supplementary file 1 [file ijms-23-05317-s001.zip › ijms-1650484-supplementary.pdf]

Supplementary Materials for Article

Impact of Adipose-Derived Mesenchymal Stem Cells (ASCs) of Rheumatic Disease Patients on T Helper Cell Differentiation

Ewa Kuca-Warnawin [1,*], Magdalena Plebańczyk [1], Marzena Ciechomska [1], Marzena Olesińska [2], Piotr Szczęsny [2] and Ewa Kontny [1]

[1] Department of Pathophysiology and Immunology, National Institute of Geriatrics, Rheumatology and Rehabilitation, Spartańska 1, 02-637 Warsaw, Poland; magdalena.plebanczyk@spartanska.pl (M.P.); marzena.ciechomska@gmail.com (M.C.); ewa.kontny@spartanska.pl (E.K.)

[2] Clinic of Connective Tissue Diseases, National Institute of Geriatrics, Rheumatology and Rehabilitation, 02-637 Warsaw, Poland; marzena.olesinska@spartanska.pl (M.O.); piotr.szczesny@spartanska.pl (P.S.)

* Correspondence: ewa.kuca-warnawin@spartanska.pl

Table S1. Expression of transcription factors, cytokines and Tregs in resting and activated T cells and PBMCs

	**$CD4^+$ T cells**			**PBMCs**			**$CD4^+$ T cells vs PBMCs** P value	
	Resting	Activated	P value	Resting	Activated	P value	Resting	Activated
**Transcription factors** (mRNA)$^{\$}$								
T-bet	0.86±0.09	1.72±0.07	***	2.0±0.14	1.026±0.02	****	###	###
GATA3	1.73±0.31	1.3±0.2	*	1.625±0.06	1.006±0.04	****	ns	ns
RORc	1.68±0.16	2.6±0.66	ns	0.773±0.06	1.034±0.13	****	##	ns
FoxP3	2.16±0.5	0.8±0.02	ns	1.675±0.18	1.183±0.097	*	ns	ns
R1	0.28±0.04	7.2±0.7	***	1.218±0.076	1.104±0.07	ns	###	###
R2	1.77±0.23	0.47±0.1	***	1.45±0.19	1.047±0.15	ns	ns	ns
**Cytokines (pg/ml)**								
IFN◎	16.5±4.05	3756±237	***	210.4±117	918±153	***	ns	###
IL-4	0.48±0.06	79.3±8.6	***	0±0	3.05±0.7	***	###	###
IL-17AF	0±0	642±130	***	0±0	256±24	***	ns	ns
**Treg cells (%)**	0.7±0.21	23.8±1.9	***	1.98±0.25	3.43±0.12	ns	ns	###

The values are the mean ± SEM; */$^{\#}P$ = 0.05-0.01, **/$^{\#\#}P$ = 0.01-0.001, ***/$^{\#\#\#}$P = 0.001-0.0001, ****/$^{\#\#\#\#}$P < 0.0001;

$^{\$}$ - relative quantification; R 1- T-bet/GATA3 ratio; R2 – RORc/FoxP3 ratio.
